# A novel difficult-to-segment samples focusing network for oral CBCT image segmentation

**DOI:** 10.1038/s41598-024-55522-7

**Published:** 2024-03-01

**Authors:** Fengjun Hu, Zeyu Chen, Fan Wu

**Affiliations:** 1 College of Information Science and Technology, Zhejiang Shuren University, Hangzhou, 310015 China; 2 Zhejiang-Netherlands Joint Laboratory for Digital Diagnosis and Treatment of Oral Diseases, Zhejiang Shuren University, Hangzhou, 310015 China

**Keywords:** Deep learning, Images segmentation, Medical image processing, Convolutional neural networks, Engineering, Mathematics and computing

## Abstract

Using deep learning technology to segment oral CBCT images for clinical diagnosis and treatment is one of the important research directions in the field of clinical dentistry. However, the blurred contour and the scale difference limit the segmentation accuracy of the crown edge and the root part of the current methods, making these regions become difficult-to-segment samples in the oral CBCT segmentation task. Aiming at the above problems, this work proposed a Difficult-to-Segment Focus Network (DSFNet) for segmenting oral CBCT images. The network utilizes a Feature Capturing Module (FCM) to efficiently capture local and long-range features, enhancing the feature extraction performance. Additionally, a Multi-Scale Feature Fusion Module (MFFM) is employed to merge multiscale feature information. To further improve the loss ratio for difficult-to-segment samples, a hybrid loss function is proposed, combining Focal Loss and Dice Loss. By utilizing the hybrid loss function, DSFNet achieves 91.85% Dice Similarity Coefficient (DSC) and 0.216 mm Average Symmetric Surface Distance (ASSD) performance in oral CBCT segmentation tasks. Experimental results show that the proposed method is superior to current dental CBCT image segmentation techniques and has real-world applicability.

## Introduction

Cone beam computed tomography (CBCT) technology is a relatively new 3D imaging technology that has been applied in clinical practice since the beginning of this century. Oral CBCT technology reorganizes multiple two-dimensional images taken by X-rays through the oral cavity to form accurate three-dimensional image data, providing more comprehensive and accurate information for dental diagnosis and treatment^1,2^. Oral CBCT technology segments the teeth in CBCT images and constructs three-dimensional models of the teeth, which can provide comprehensive access to tooth position and tooth morphology, etc., and help dentists assess the morphological characteristics of diseases such as dental relationships, maxillofacial deformities, and oral diseases. In summary, CBCT technology and the corresponding constructed 3D dental models are an indispensable tool for dentists, providing a scientific, accurate and efficient solution in the diagnosis and treatment of oral diseases. However, segmentation of dental parts from CBCT images is a complex and tedious task, which is still mainly performed manually by professionals with knowledge of dental imaging^3^. This not only consumes a significant amount of time and labor cost, but also the subjective judgment of the annotator can affect the annotation results, which greatly limits the work efficiency.

With the continuous progress of deep learning technology, it has been widely used in various fields, and gradually applied to the segmentation task of oral CBCT image^4–7^. Chen et al. employed a multi-task 3D fully convolutional network and marker-controlled watershed transform to segment individual teeth^8^ Cui et al. utilized a two-stage network for generating accurate segmentation and identification results automatically^9^. Ma et al. designed a lightweight end-to-end CNN architecture using ordinary convolutions, dilated convolutions, and residual connections as basic modules for automatically segmenting teeth from CBCT images^10^.Jang et al. proposed a fully automated method for identifying and segmenting 3D individual teeth from dental CBCT images^11^. Jaskari et al. evaluated the application effect of the full convolutional deep neural network model in CBCT image segmentation task, and obtained high-quality segmentation results, proving the effectiveness of deep learning technology in CBCT image segmentation task^12^.

However, using deep learning techniques to segment oral CBCT images has its limitations. Affected by various factors such as X-ray beam angle, scanning parameters, noise, and artifacts, the tooth contours are blurred and it is difficult to identify the boundary between the tooth and the alveolar bone. Moreover, the root region occupies a smaller proportion of the image compared to the crown region, resulting in significant scale differences between different segmentation targets. These problems lead to relatively low segmentation accuracy of the crown edge and root portion compared to the interior of the crown, thus defining the crown edge and root portion as difficult segmentation samples in the oral CBCT segmentation task. In summary, a comprehensive and in-depth study of the network structure is necessary to improve the segmentation accuracy of the network for difficult-to-segment samples. The main contributions of this work are outlined as follows:

1. We propose a difficult-to-segment samples focusing network, i.e., DSFNet. The Feature Capture Modules (FCM) of the network efficiently capture both local features and long-range features to enhance the feature extraction performance of the network. In addition, the network also uses a Multi-scale Feature Fusion Module (MFFM) to fuse multi-scale feature information.
2. We propose a Mixed Loss Function that combines Focal Loss and Dice Loss, and improves it for Dice Loss. This function is dedicated to further improve the Loss ratio of hard-to-segment samples and make the network focus more on difficult-to-segment samples.
3. With the use of Mixed Loss Function, the proposed network obtained 91.85% DSC and 0.216 mm ASSD in the oral CBCT segmentation task, which is better than the existing models.

## Related works

U-Net is a well-known network utilized for medical image segmentation tasks^13^. Its U-shaped design and inclusion of skip connections allow for effective extraction and fusion of multi-scale features. Consequently, this architecture demonstrates enhanced segmentation performance and robustness in a variety of medical image segmentation tasks, such as brain^14^, lung^15^, and heart^16^. The U-Net network structure comprises two main components: an encoder and a decoder. The encoder, consisting of a convolutional network with four downsampling modules, reduces the image dimension while extracting image features. On the other hand, the decoder employs a four-layer deconvolution module for upsampling, restoring the feature map to its original resolution. Furthermore, U-Net utilizes skip connections to merge each upsampled feature map with the corresponding feature map extracted during downsampling, thereby facilitating the fusion of shallow and deep feature information.

U-Net has proven to be a simple and effective model for medical image segmentation. To further enhance its performance, recent advancements in medical image segmentation networks have built upon the structural characteristics of U-Net by introducing modifications or additional modules. One such example is Attention U-Net, which incorporates attention gates into the original skip connection^17^. This introduces an attention mechanism that allows the network to focus more on important regions within the feature map. Another improvement is seen in TransUNet^18^ and Swin-UNet^19^, where the Transformer architecture is integrated into medical image segmentation, replacing some or all of the convolution modules. This transformation enhances the network's ability to model long-range dependencies. Two commonly used image segmentation networks, SegNet^20^ and DeepLab V3+^21^, also share structural similarities with U-Net. Both employ encoder-decoder structures for downsampling and upsampling of feature maps. SegNet distinguishes itself by recording the index of the retained element in each Max pooling, allowing for accurate object boundary segmentation during upsampling. On the other hand, DeepLab V3+ utilizes Atrous Spatial Pyramid Pooling to achieve multi-scale feature extraction through different scale cavity convolutions.

Each of the traditional networks mentioned above has been enhanced based on the U-Net architecture. Despite demonstrating certain advantages in various medical image segmentation tasks, these traditional networks still exhibit shortcomings when applied to dental CBCT image segmentation. One prominent issue is the network’s inadequacy in accurately difficult-to-segment samples in dental CBCT images, particularly those with small-scale and boundary-blurred characteristics. Consequently, further optimization of the network structure is imperative, along with an improvement in its ability to process such difficult samples, in order to address this problem.

## Methodologies

### Overall architecture

**Figure 1:**
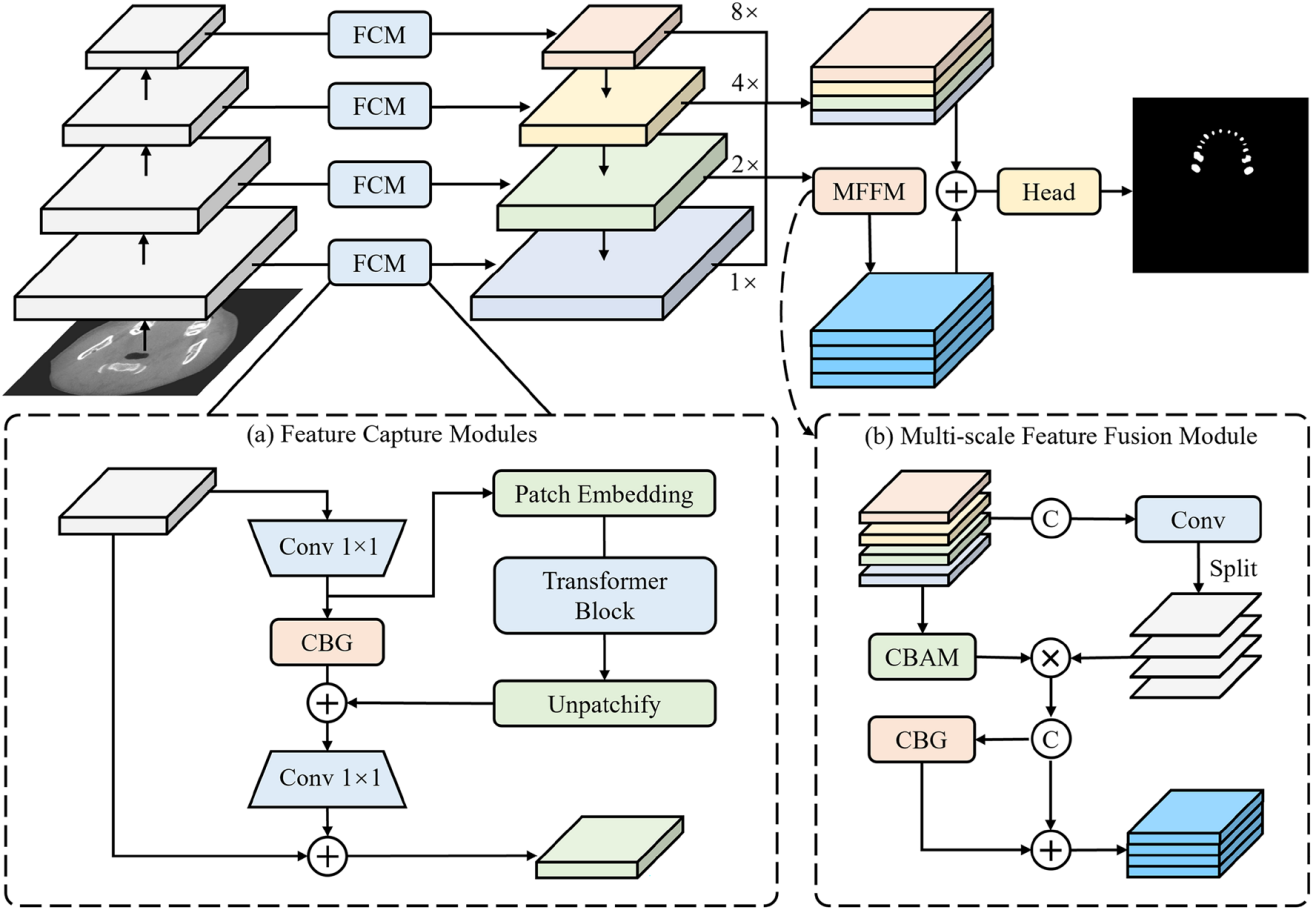
Network structure of DSFNet.

We propose a novel network, DSFNet, for the segmentation of oral CBCT images, as illustrated in Fig. 1. This network incorporates the U-shaped structure, along with skip connections, featuring three downsampling and three upsampling stages. To enhance the overall performance of the model, we introduce the Feature Capture Module (FCM) within the skip connections. The FCM extracts both local and long-range feature representations simultaneously using both convolution and transformer operations. Additionally, we introduce the Multi-scale Feature Fusion Module (MFFM), which is placed before the segment head. The MFFM effectively fuses multi-scale feature information using the attention mechanism, thereby reducing the noise caused by skip connections. This module addresses the challenges imposed by scale differences on difficult-to-segment samples. Furthermore, to assign higher weight to difficult-to-segment samples, we propose a new loss function that combines dice loss and focal loss.

### Feature capture module

To enhance the performance of the network, a Feature Capture Module (FCM) is proposed in this work, which efficiently captures both local features and long-range features to enhance the feature extraction performance. The structure of this module is illustrated in Fig. 1a.

The proposed Feature Capture Module consists of two parts: the convolution block and the Transformer block, which respectively extract local and global features. The convolution block adopts a bottleneck structure. First, a convolution with a kernel size of 1 × 1 is used to reduce the channel dimension to one-fourth of the original channels. Then, the CBG module (Conv-BN-GELU) is employed to extract features. Finally, another convolution with a kernel size of 1 × 1 is applied to restore the channel dimension of the feature maps. The operations of the CBG module can be represented as:

1 $${\text{CBG}}(F) = {\text{GELU}}({\text{BN}}(f^{3 \times 3} (F)))$$

where $f^{3 \times 3}$ represents a convolution operation with a kernel size of 3 ×3. The calculation of the Transformer block is as follows:

2 $$\begin{gathered} F = [F_{p1} ;F_{p2} ; \ldots ;{\text{F}}_{pN} ] \hfill \\ z_{0} = [F_{p1} {\text{E}};F_{p2} {\text{E}}; \ldots ;{\text{x}}_{pN} {\text{E}}]{\text{ + E}}_{{{\text{pos}}}} \hfill \\ z^{\prime } = {\text{MSA}}({\text{LN}}({\text{z}}_{0} )) + z_{0} \hfill \\ F_{trans} = {\text{MLP}}({\text{LN}}({\text{z}}^{\prime } )) + z^{\prime } \hfill \\ \end{gathered}$$

where ${\text{E}}$ is the projection matrix, ${\text{E}}_{{{\text{pos}}}}$ is the positional encoding, ${\text{MSA}}( \cdot )$ indicates the multi-head self-attention operation, and ${\text{LN}}( \cdot )$ represents layer normalization. Due to the heavy computational cost of self-attention operations, this work utilizes the compressed feature maps in the bottleneck for computation. The calculation of the FCM's output, denoted as $F_{out}$, given the input $F$, is defined as:

3 $$\begin{gathered} F^{\prime } = f_{c/4}^{1 \times 1} (F) \hfill \\ F_{out} = F + f_{c \times 4}^{1 \times 1} ({\text{CBG}}(F^{\prime } ) + F_{trans} ) \hfill \\ \end{gathered}$$

where $f_{c/4}^{1 \times 1}$ represents a convolution operation with a kernel size of $1 \times 1$ and output channels quarter of the input channels, $f_{c \times 4}^{1 \times 1}$ represents a convolution operation with a kernel size of and output channels four times of the input channels, and $F_{trans}$ is calculated according to Eq. (2).

### Multi-scale feature fusion module

In order to alleviate the impact of scale differences on segmentation accuracy, a Multi-scale Feature Fusion Module is proposed in this work, which utilizes attention mechanisms to construct intra-scale attention and inter-scale attention, thus enhancing the segmentation effect on challenging samples. The structure of this module is illustrated in Fig. 1b.

The input of this module is different scale feature map that have been interpolated to the same resolution, denoted as $F_{i}$ and $i = 1,2,3,4$. The MFFM have two pathways, the first pathway utilizes the Convolutional Block Attention Module (CBAM) to process the feature maps of each scale, constructing intra-scale attention to make the network focus more on important regions within the feature maps and obtain intra-scale attention features. This operation is defined as:

4 $$\begin{gathered} {\text{CBAM}}(F_{i} ) = (F_{i} \odot {\text{M}}_{c} (F_{i} )) \odot ({\text{M}}_{s} (F_{i} \odot {\text{M}}_{c} (F_{i} ))) \hfill \\ {\text{where}}\;{\kern 1pt} i = 1,2,3,4 \hfill \\ {\text{M}}_{c} (F) = \sigma ({\text{MLP(AvgPool}}_{c} (F){\text{) + MLP(MaxPool}}_{c} (F){)}) \hfill \\ {\text{M}}_{s} (F) = \sigma (f^{7 \times 7} {\text{([AvgPool}}_{s} (F){\text{;MaxPool}}_{s} (F)])) \hfill \\ \end{gathered}$$

where ${\text{AvgPool}}_{c} ( \cdot )$ and ${\text{MaxPool}}_{c} ( \cdot )$ denote as average pooling and max pooling along the channel dimension, while ${\text{AvgPool}}_{s} ( \cdot )$ and ${\text{MaxPool}}_{s} ( \cdot )$ are along the spatial dimension, $f^{7 \times 7} ( \cdot )$ denote as the convolution operation with a kernel size of $7 \times 7$, and ${\text{MLP(}} \cdot {)}$ is the multi-layer perceptron with shared parameters, $\odot$ denotes Hadamard product.

In the other pathway, different scale features are concatenated along the channel dimension and then processed with a convolution operation to construct inter-scale attention, resulting in attention features with a channel dimension of 4. The attention features in each channel are multiplied with the intra-scale attention features obtained from the first pathway, and the concatenated results are fused using the CBG module to integrate features from different scales. The calculation is defined as:

5 $$\begin{gathered} {\text{MFFM}}(F_{i} ) = {\text{CBG}}(F_{c} ) + F_{c} \hfill \\ F_{c} = [F_{1}^{\prime } ;\;F_{2}^{\prime } ;\;F_{3}^{\prime } ;\;F_{4}^{\prime } ] \hfill \\ F_{i} ^{\prime} = {\text{CBAM}}(F_{i} ) \odot A_{i} \hfill \\ [A_{1} ;\;A_{2} ;\;A_{3} ;\;A_{4} ] = f_{c = 4}^{7 \times 7} ([F_{1} ;\;F_{2} ;\;F_{3} ;\;F_{4} ]) \hfill \\ \end{gathered}$$

where $f_{c = 4}^{7 \times 7}$ represents the output feature map with a channel size of 4 and a convolutional kernel size of $7 \times 7$. The MFFM effectively integrates features from different scales, further improving the model performance.

### Mixed loss function

The boundaries of teeth and alveolar bone in oral CBCT images are blurred and difficult to identify, and the proportion of teeth and background occupying the image varies greatly. Therefore, the traditional cross-entropy loss function often fails to achieve good segmentation results. In order to solve the above problems, a Mixed Loss Function combining Focal Loss and Dice Loss is proposed in this work, as in

6 $${\mathcal{L}\ominus } = \alpha {\mathcal{L}}_{{D^{\prime } }} + (1 - \alpha ){\mathcal{L}}_{F}$$

where ${\mathcal{L}}_{{D^{\prime } }}$ denotes the improved Dice Loss, ${\mathcal{L}}_{F}$ denotes Focal Loss, and $\alpha$ is the scale factor whose value range is [0,1]. The calculation method of Focal Loss is shown in Eq. (7) below:

7 $${\mathcal{L}}_{F} (y_{t} ) = - (1 - y_{t} )^{\gamma } \log (y_{t} )$$

where $\gamma$ denotes to the relative loss of a moderator due to increasing the number of hard-to-classify samples. $y_{t}$ is calculated as follows:

8 $$y_{t} = \left\{ {\begin{array}{*{20}c} {\hat{y},} & {y = 1} \\ {1 - \hat{y},} & {y = 0} \\ \end{array} } \right.$$

where $y$ and $\hat{y}$ denote the ground truth and predicted value. Dice Loss is a loss function commonly used to solve the problem of positive and negative sample imbalance in segmentation tasks, which is calculated as follows:

9 $${\mathcal{L}}_{D} (y) = 1 - \frac{{2\hat{y} \cdot y + \epsilon }}{{\hat{y} \cdot y + y + \epsilon }}$$

where $\epsilon$ denotes the smoothing factor. Based on the idea of Focal Loss, this study improves Dice Loss to make it more focused on hard-to-segment samples, which is calculated as follows:

10 $${\mathcal{L}}_{D*} (y) = 1 - \frac{{2^{(1 + \gamma ^{\prime})} \hat{y} \cdot y + \epsilon }}{{(\hat{y} \cdot y + y)^{(1 + \gamma ^{\prime})} + \epsilon }}$$

**Figure 2:**
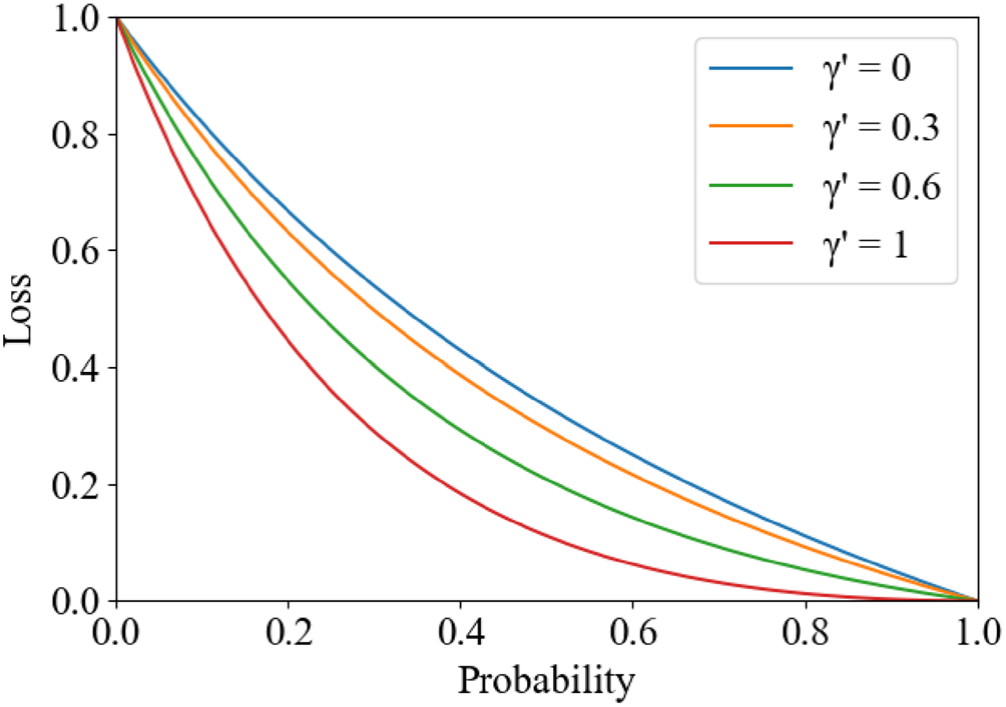
Schematic diagram of the effect of the loss function of the improved Dice Loss.

The $\gamma^{\prime } \in [0,1]$ also acts as a regulator of improved Dice Loss. When $\gamma^{\prime } \in (0,1]$, the loss of the more easily segmented samples will be less, and the ratio of the loss of hard-to-classify samples and easy-to-segment samples will increase, making the loss function more inclined to hard-to-segment samples and helping to improve the accuracy of segmentation of hard-to-segment samples. When $\gamma^{\prime } = 0$, the loss function degenerates to the original Dice Loss function. The effect of this improved loss function of Dice Loss is shown schematically in Fig. 2.

As an example, for sample $\hat{y} = 0.8$ and sample $\hat{y} = 0.5$, in the case of $\gamma^{\prime } = 0$ (the original Dice Loss), ${\mathcal{L}}_{{D,\hat{y} = 0.8}} = 0.111$ and ${\mathcal{L}}_{{D,\hat{y} = 0.5}} = 0.333$. The Loss ratio for both is 1:3. In the case of $\gamma^{\prime } = 1$, ${\mathcal{L}}_{{D*,\hat{y} = 0.8}} = 0.012$ and ${\mathcal{L}}_{{D,\hat{y} = 0.5}} = 0.111$. The Loss ratio of the two samples is about 1:9. It can be seen that the ratio of difficult samples will be higher with the improved Dice Loss, and the model will focus more on reducing the loss of difficult samples during the training process.

## Experiments

### Dataset and experiment details

The dataset contains 150 patients’ CBCT images and corresponding annotations^22^.For data preprocessing, the dataset was processed in this study to standardize the scale of the input CBCT images. First, the voxel size of all CBCT data was uniformly resampled to 0.4 mm, and the 98 sets of CBCT images with higher resolution were selected and constructed as the experimental dataset. To mitigate the impact of metal artifacts, the intensity values of each CBCT scan were cropped to [0,2500]. Subsequently, the voxel intensities were then normalized to the range of [0,1] following the standard protocol for image processing in deep learning. In addition, the original dataset is used for the instance segmentation task, and the binary classification task studied in this work involves the segmentation of teeth and background. Therefore, this study modifies the categories in the label to represent the background region with 0 and the teeth region with 1. Finally, the processed 98 datasets are divided into training set, validation set, and test set in a 6:2:2 ratio. The processed dataset of this work can be obtained by contacting the corresponding author.

In terms of experimental details, four NVIDIA A100 GPUs were used for training, the input image size was set to $224\; \times \;224$, and the batch size was set to 128. The initial learning rate was set to 3e−4, and 50 epochs were trained using the AdamW optimizer with a weight decay of 0.05, while the learning rate was gradually reduced using the cosine learning rate scheduler to ensure convergence of each model. In this study, the model's performance of the model was verified using the validation set after each epoch, and the optimal weight parameters were saved and tested on the test set to obtain the final accuracy of segmentation.

Regarding the evaluation indices of segmentation, this study also adopts Dice Similarity Coefficient (DSC) and Average symmetric surface distance (ASSD) as evaluation indexes of segmentation results. DSC can better reflect the segmentation accuracy within the target region, while ASSD can better reflect the segmentation accuracy of the target boundary.

### Comparison experiment

**Figure 3:**
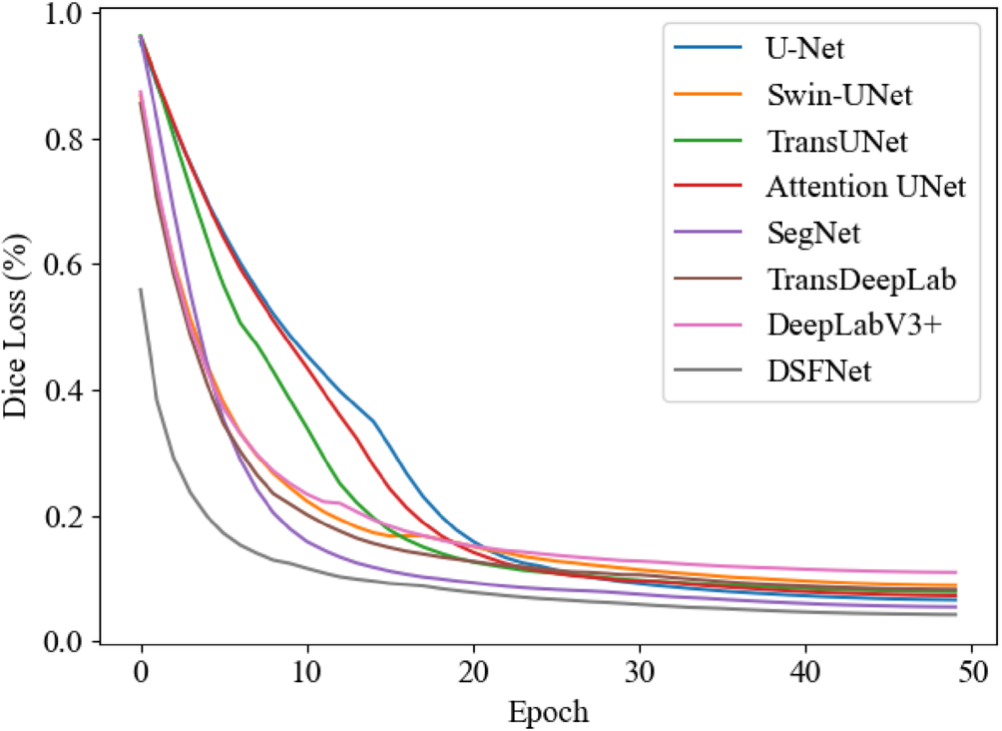
Train loss curve.

**Figure 4:**
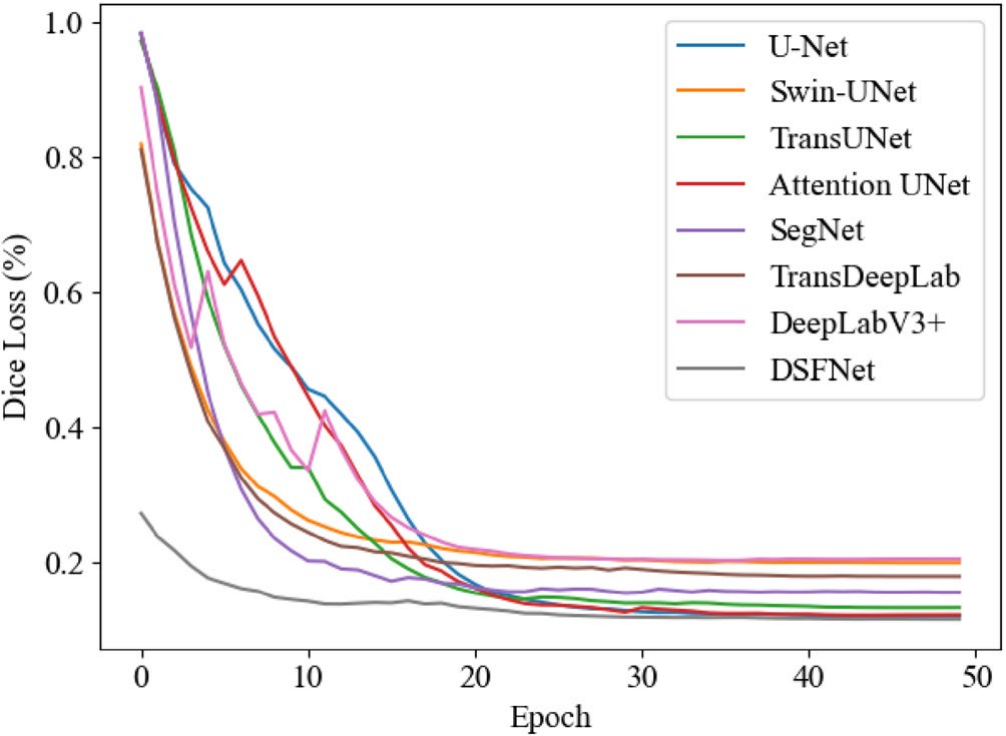
Validation loss curves smoothed using exponentially weighted averaging, $\beta \;{ = }\;0.7$.

To test the effectiveness of DSFNet, firstly, it was trained on the oral CBCT image dataset and compared with the existing medical image segmentation network. The traditional Dice Loss was used for the loss function. the training loss and validation loss during training are shown in Figs. 3 and 4.

As shown in the above figures, after 50 epochs of training, all the trained models have converged. Under the comparison of the same training parameters, the DSFNet proposed in this work converges faster and the loss difference between the training and validation sets is lower, indicating that DSFNet has better generalization performance. The segmentation results of each network are shown in Table 1.

**Table 1:** Performances of different network.

Method	DSC (%)	ASSD (mm)
U-Net	89.47	0.314
Attention UNet	90.73	0.272
SegNet	89.10	0.287
DeepLab V3+	82.88	0.831
TransDeepLab	86.58	0.459
TransUNet	90.61	0.257
Swin-UNet	84.91	0.435
DSFNet (Ours)	91.31	0.249

**Figure 5:**
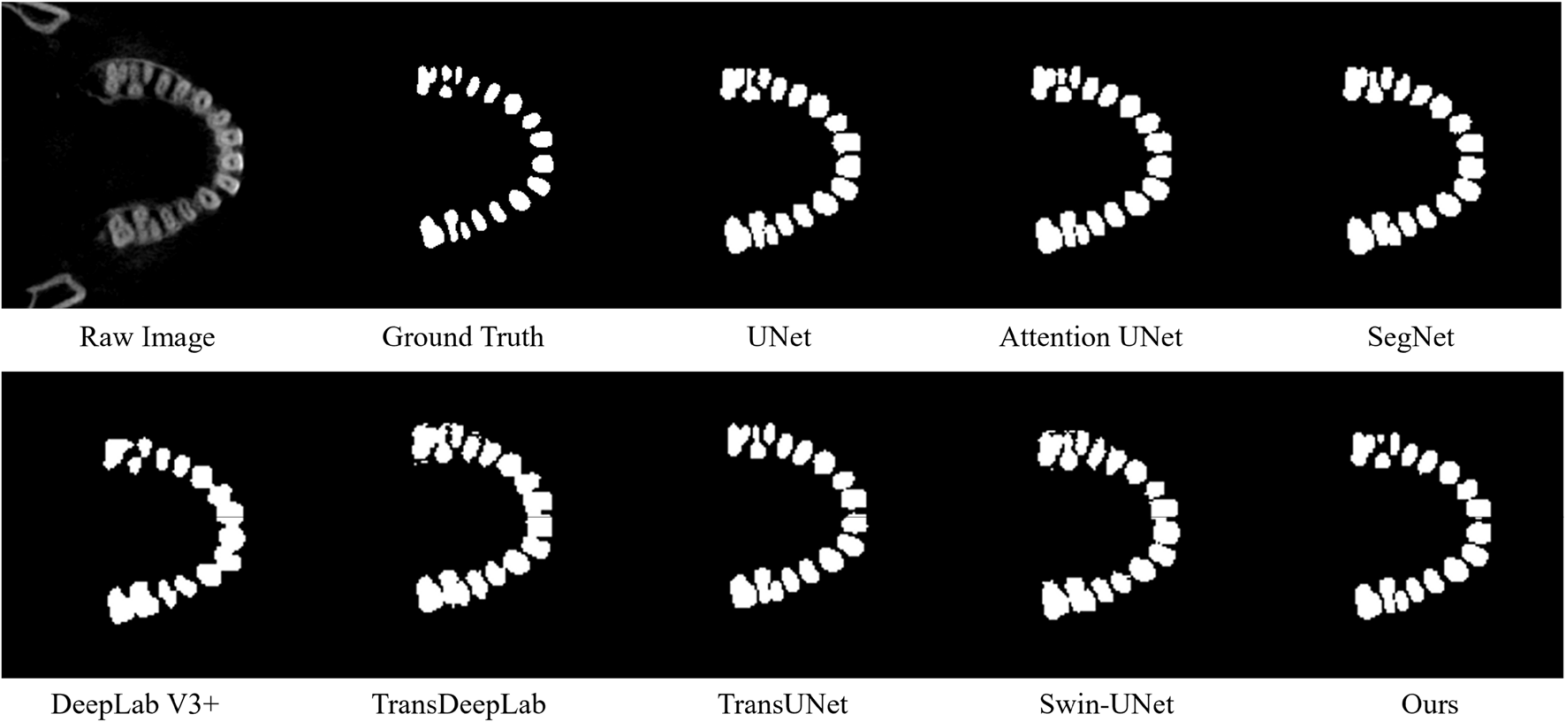
The visualization of the segmentation results used different network.

The segmentation results showed that the improved DSFNet model proposed in this study achieved a DSC score of 91.31% and an ASD of 0.249 mm, and the segmentation accuracy inside the tooth region and at the boundary was better than that of the existing model. The visualization of the segmentation results is shown in Fig. 5.

**Figure 6:**
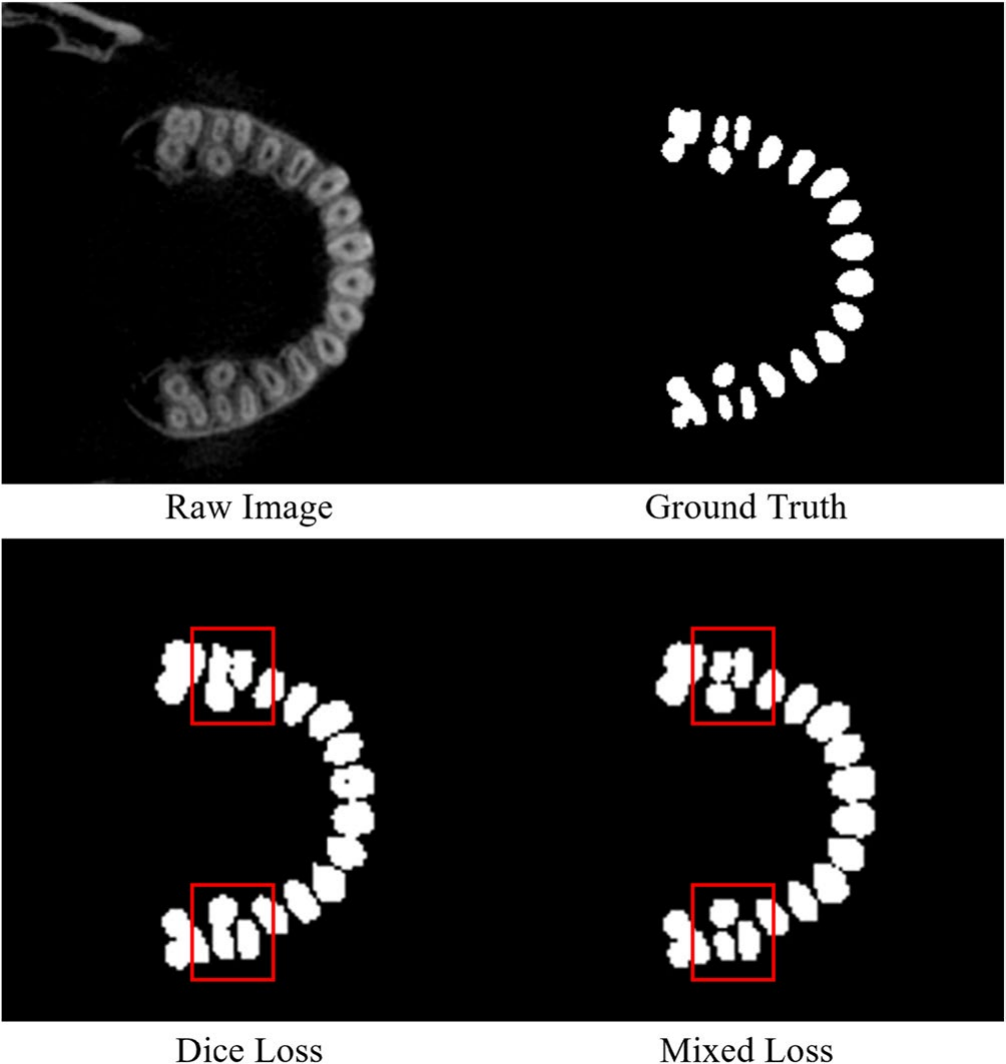
The visualization of the segmentation results used different loss function.

To verify the effectiveness of the Mixed Loss Function proposed in this work, set the conditioning factor $\gamma = 2$ of Focal Loss, and DSFNet is trained under the Mixed Loss Function scaling factor $\alpha \in \{ 0,\;0.5,\;1\}$ and the conditioning factor $\gamma^{\prime } \in \{ 0,\;0.5,\;1\}$ of the improved Dice Loss, respectively. The training results are shown in Table 2.The training results show that DSFNet can achieve the highest accuracy with $\alpha { = }\;1$ and $\gamma^{\prime } { = }\;1$, which proves the effectiveness of Mixed Loss Function. The visual segmentation results of DSFNet trained by Dice Loss and Mixed Loss Function with $\alpha = 0.5$, $\gamma^{\prime } = 1$ are shown in Fig. 6.

**Table 2:** Performance under different loss function parameter.

$\alpha$	$\gamma^{\prime }$	DSC (%)	ASSD (mm)
0	–	90.10	0.252
0.5	0	91.39	0.237
0.5	0.5	91.56	0.223
0.5	1	91.85	0.216
1	0	91.31	0.249
1	0.5	91.43	0.237
1	1	91.41	0.231

### Ablation experiment

To verify the importance of each module in DSFNet, a set of ablation studies were conducted in this study. Firstly, FCM and MFFM in DSFNet were eliminated, and a baseline was trained using the original Dice loss function, as shown in Table 3.

**Table 3:** Results of ablation experiment.

NO	FCM	MFFM	Mixed loss function	DSC (%)	ASSD (mm)
1	×	×	×	89.33	0.346
2	√	×	×	90.14	0.285
3	×	√	×	90.48	0.263
4	√	√	×	91.31	0.249
5	√	√	√	91.85	0.216

Based on the baseline, FCM and MFFM were added for training and compared, as shown in NO. 1–3, respectively. The experimental results show that both FCM and MFFM can effectively improve the network performance. In addition, this study further compares the effect of using Mixed Loss Function, as shown in NO. 4–5. The experimental results show that the Loss Function can further improve the performance of the model after training and improves the DSC by 2.52% and the ASSD by − 0.13 mm compared to the baseline.

## Conclusion

This work provides an in-depth study of the current challenges in segmenting oral CBCT images using deep neural networks and introduces DSFNet, a novel architecture tailored for difficult-to-segment samples. The network leverages FCM to efficiently extract both local and long-range features, while the MFFM integrates information across various scales. This dual approach significantly enhances the network’s ability to process challenging samples. Additionally, we introduce a Mixed Loss Function designed to improve the segmentation performance on these hard-to-segment samples by optimizing the loss ratio during training. The experimental findings demonstrate that DSFNet surpasses existing models, achieving a 91.85% DSC in dental CBCT image segmentation, particularly excelling in the segmentation of difficult samples.

## Data Availability

The data used to support the findings of this study are available from the corresponding author upon request.
